# Supplementary material for: Pyruvate Dehydrogenase Kinase 1 inhibition mediated oxidative phosphorylation enhancement in cartilage promotes osteoarthritis progression
Source: BMC Musculoskelet Disord. 2023 Jul 20;24:597. doi: 10.1186/s12891-023-06585-6 (PMC10357736; doi:10.1186/s12891-023-06585-6)
Supplement: Supplementary file 1 — Additional file 1. [file 12891_2023_6585_MOESM1_ESM.zip › MF.Gsea.1653627316242/GOMF_FATTY_ACID_DERIVATIVE_BINDING.html]

Details for gene set GOMF\_FATTY\_ACID\_DERIVATIVE\_BINDING[GSEA]

|  || Dataset | 55457\_collapsed\_to\_symbols.cls.cls #OA\_versus\_normal.cls.cls #OA\_versus\_normal\_repos |
| Phenotype | cls.cls#OA\_versus\_normal\_repos |
| Upregulated in class | OA |
| GeneSet | GOMF\_FATTY\_ACID\_DERIVATIVE\_BINDING |
| Enrichment Score (ES) | 0.44045937 |
| Normalized Enrichment Score (NES) | 1.3221896 |
| Nominal p-value | 0.13304721 |
| FDR q-value | 0.38663563 |
| FWER p-Value | 1.0 |
Table: GSEA Results Summary

  

Fig 1: Enrichment plot: GOMF\_FATTY\_ACID\_DERIVATIVE\_BINDING      
 Profile of the Running ES Score & Positions of GeneSet Members on the Rank Ordered List

  

| SYMBOL | TITLE | RANK IN GENE LIST | RANK METRIC SCORE | RUNNING ES | CORE ENRICHMENT || 1 | SCP2 | sterol carrier protein 2 [Source:HGNC Symbol;Acc:HGNC:10606] | 105 | 0.905 | 0.2217 | Yes |
| 2 | ECI2 | enoyl-CoA delta isomerase 2 [Source:HGNC Symbol;Acc:HGNC:14601] | 669 | 0.601 | 0.3306 | Yes |
| 3 | ACOT7 | acyl-CoA thioesterase 7 [Source:HGNC Symbol;Acc:HGNC:24157] | 971 | 0.524 | 0.4405 | Yes |
| 4 | HADHA | hydroxyacyl-CoA dehydrogenase trifunctional multienzyme complex subunit alpha [Source:HGNC Symbol;Acc:HGNC:4801] | 4163 | 0.141 | 0.2287 | No |
| 5 | ACBD4 | acyl-CoA binding domain containing 4 [Source:HGNC Symbol;Acc:HGNC:23337] | 4880 | 0.082 | 0.1938 | No |
| 6 | ALDH6A1 | aldehyde dehydrogenase 6 family member A1 [Source:HGNC Symbol;Acc:HGNC:7179] | 5560 | 0.028 | 0.1482 | No |
| 7 | ACADVL | acyl-CoA dehydrogenase very long chain [Source:HGNC Symbol;Acc:HGNC:92] | 6394 | -0.034 | 0.0922 | No |
| 8 | ACADL | acyl-CoA dehydrogenase long chain [Source:HGNC Symbol;Acc:HGNC:88] | 6427 | -0.037 | 0.0992 | No |
| 9 | SOAT2 | sterol O-acyltransferase 2 [Source:HGNC Symbol;Acc:HGNC:11178] | 6750 | -0.059 | 0.0893 | No |
| 10 | SOAT1 | sterol O-acyltransferase 1 [Source:HGNC Symbol;Acc:HGNC:11177] | 7555 | -0.124 | 0.0583 | No |
| 11 | ACOT11 | acyl-CoA thioesterase 11 [Source:HGNC Symbol;Acc:HGNC:18156] | 7746 | -0.137 | 0.0785 | No |
| 12 | DBI | "diazepam binding inhibitor, acyl-CoA binding protein [Source:HGNC Symbol;Acc:HGNC:2690]" | 7929 | -0.149 | 0.1023 | No |
| 13 | GCDH | glutaryl-CoA dehydrogenase [Source:HGNC Symbol;Acc:HGNC:4189] | 8180 | -0.170 | 0.1259 | No |
| 14 | PNPLA3 | patatin like phospholipase domain containing 3 [Source:HGNC Symbol;Acc:HGNC:18590] | 8455 | -0.190 | 0.1530 | No |
| 15 | PITPNA | phosphatidylinositol transfer protein alpha [Source:HGNC Symbol;Acc:HGNC:9001] | 8883 | -0.223 | 0.1766 | No |
| 16 | ACBD3 | acyl-CoA binding domain containing 3 [Source:HGNC Symbol;Acc:HGNC:15453] | 11697 | -0.532 | 0.0934 | No |
Table: GSEA details [plain text format]

  

Fig 2: GOMF\_FATTY\_ACID\_DERIVATIVE\_BINDING      
 Blue-Pink O' Gram in the Space of the Analyzed GeneSet

  

Fig 3: GOMF\_FATTY\_ACID\_DERIVATIVE\_BINDING: Random ES distribution      
 Gene set null distribution of ES for **GOMF\_FATTY\_ACID\_DERIVATIVE\_BINDING**
